# Comparative Transcriptome Analysis Reveals the Potential Cardiovascular Protective Targets of the Thyroid Hormone Metabolite 3-Iodothyronamine (3-T1AM)

**DOI:** 10.1155/2020/1302453

**Published:** 2020-06-19

**Authors:** Zhou Haiyan, Hu Bailong, Zhang Bei, Wang Yiming, Liu Xingde

**Affiliations:** ^1^Guizhou Medical University, 550004 Guiyang, China; ^2^Clinical Research Centre, The Affiliated Hospital of Guizhou Medical University, 550004 Guiyang, China; ^3^Department of Anesthesiology, The Affiliated Hospital of Guizhou Medical University, Guiyang, Guizhou Province 550004, China; ^4^Department of Psychology, The Affiliated Hospital of Guizhou Medical University, Guiyang, Guizhou Province 550004, China; ^5^Department of Cardiology, The Second Affiliated Hospital of Guizhou University of Traditional Chinese Medicine, 550004 Guiyang, China

## Abstract

**Background:**

The thyroid hormone metabolite 3-iodothyronamine (3-T1AM) is rapidly emerging as a promising compound in decreasing the heart rate and lowering the cardiac output. The aim of our study was to fully understand the molecular mechanism of 3-T1AM on cardiomyocytes and its potential targets in cardiovascular diseases.

**Materials and Methods:**

In our study, we utilized RNA-Seq to characterize the gene expression in H9C2 cells after 3-T1AM treatment. Comparative transcriptome analysis, including gene ontology, signaling pathways, disease connectivity analysis, and protein-protein interaction networks (PPI), was presented to find the critical gene function, hub genes, and related pathways.

**Results:**

A total of 1494 differently expressed genes (DEGs) were identified (192 upregulated and 1302 downregulated genes) in H9C2 cells for 3-T1AM treatment. Of these, 90 genes were associated with cardiovascular diseases. The PPI analysis indicated that 5 hub genes might be the targets of 3-T1AM. Subsequently, eight DEGs characterized using RNA-Seq were confirmed by RT-qPCR assays.

**Conclusions:**

Our study provides a comprehensive analysis of 3-T1AM on H9C2 cells and delineates a new insight into the therapeutic intervention of 3-T1AM for the cardiovascular diseases.

## 1. Introduction

3-Iodothyronamine (3-T1AM) is a metabolite product of the thyroid hormones (TH) which exerts an opposite physiological function of classical TH such as a decrease of body temperature and metabolic rate in rodents [1]. Several studies have shown that 3-T1AM is presumed to act on various organs and different receptors rapidly [2]. Moreover, it has therapeutic potential for treatment of metabolic diseases [3], myocardial vascular diseases [4], and neurological diseases [5].

Thyroid hormones and their metabolite products play a critical role in the development of cardiovascular diseases [6–8]. Of note, 3-T1AM, a decarboxylated and deiodinated thyroid hormone derivative, has displayed a potency to decrease the heart rate and lower the cardiac output [9], but the underlying mechanism between 3-T1AM and myocardial vascular diseases has remained unknown. It would therefore be of interest to perform RNA-Seq assay to predict the potential transcriptional mechanism used by 3-T1AM to protect from the myocardial injuries. In our present study, we aim to identify differently expressed genes in H9C2 cells and cells treated with 3-T1AM and investigate the underlying molecular mechanism of its effect on myocardial protection.

## 2. Materials and Methods

### 2.1. Cell Cultures

H9C2 cells were purchased from ATCC. H9C2 cells were cultured in DMEM (Gibco) supplemented with 10% fetal bovine serum (FBS, Gibco), penicillin (100 U/ml, Gibco), and streptomycin (100 U/ml, Gibco) in a humidified cell incubator providing 5% CO2/95% fresh air at 37°C. Cells were exposed to 20 *μ*M 3-T1AM for 24-hour treatment. H9C2 cells cultured in 3-T1AM-free growth medium were used as controls.

### 2.2. Cell Viability Assessments

The cell viability was assessed by CCK-8 assay. Briefly, cells were seeded into a 96-well culture plate at a density of 6 × 10^3^/well. Cells were treated with various doses of 3-T1AM for 24 h. H9C2 cells cultured in 3-T1AM-free growth medium were used as controls. Then, cells in each well were incubated with CCK-8 at 37°C for 4 hours. The absorbance was determined by a plate reader.

### 2.3. Total RNA Extraction

Total RNA in both groups (control and 3-T1AM treatment) was extracted with RNAiso Plus (Takara) according to the manufacturer's instructions. The cDNA library was constructed based on the instructions of Illumina. RNA-Seq of each samples was performed by Sangon Biotech Co. Ltd. (Shanghai). Methods of RNA-Seq analysis were described in previous studies [10].

### 2.4. Construction of RNA-Seq Library for Illumina Sequencing

Sequencing library preparations were constructed using the manufacturer's protocol (VAHTSTM mRNA-seq V2 Library Prep Kit for Illumina®). The digested RNA samples were used for first- and second-strand cDNA synthesis with random hexamer primers. After the first- and second-strand cDNA synthesis, the double-stranded products were end repaired, a single “A” base was added, and adaptors were ligated onto the cDNA products. cDNA libraries were constructed. Then, the paired-end sequencing was used on an Illumina HiSeq™ 2500 [11].

### 2.5. Identification of Differentially Expressed Genes (DEGs)

After sequencing, raw data were obtained in the fastq format. FastQC was used for validating the quality of the data. Trimming of sequences was performed using Trimmomatic. Then, quality check using FastQC was performed again on the trimmed sequences. Reads per kilobase of exon mode per million mapped reads (RPKM) were employed to quantify transcript levels [12]. Differentially expressed genes in four samples were sought, and conditions for differences in genes were filtered. The adjusted *P* value < 0.05 and Log2(fold change) > 1 were set as the cutoff criterion.

### 2.6. Gene Ontology and KEGG Pathway Enrichment Analysis

The Database for Annotation, Visualization and Integrated Discovery (DAVID 6.8) gene annotation tool (http://david.ncifcrf.gov/tools.jsp) was used to perform GO and KEGG analysis [13]. Gene ontology term analyses of the DEGs, including biological process (BP), cellular component (CC), and molecular function (MF), were identified using the gene ontology (GO) project (http://www.geneontology.org). KEGG pathway enrichment analysis was performed based on the Kyoto Encyclopedia of Genes and Genomes database (http://www.kegg.jp) [14]. In addition, GO terms and pathway with *P* values less than 0.05 were considered to be statistically significant.

### 2.7. Construction of Protein-Protein Interaction (PPI) Network

The PPI network was generated based on STRING 10.0 (http://String-db.org) [15]. A confidence score of 0.9 was set as the cutoff criterion. All of the networks were visualized and analyzed with Cytoscape 3.4.0 (http://cytoscape.org). Moreover, the significant network modules with degree cutoff = 2, node score cutoff = 0.2, and *k* − score = 2 were constructed using the Molecular Complex Detection (MCODE) plugin for Cytoscape.

### 2.8. Disease Connectivity Analysis

Diseases enriched with 3-T1AM-treated associated genes were identified using the Comparative Toxicogenomics Database (Bonferroni-corrected *P* value < 0.05).

### 2.9. Quantitative Real-Time PCR (RT-qPCR)

In order to confirm the DEGs of RNA-Seq, the expression levels of 8 genes, such as Camk2d, Ppp2Ca, Nf1, Yes1, Psma6, Psmb6, Jak2, and Sirt4, were chosen and analyzed by RT-qPCR. Total RNA in both groups (control and 3-T1AM treatment) was extracted with RNAiso Plus (Takara) according to the manufacturer's instructions. RT-qPCR was conducted by using the Vazyme HiScript II One Step qRT-PCR SYBR Green Kits (Vazyme, Nanjing) on a 7500 real-time PCR system (ThermoFisher Scientific). GAPDH was used as a control for normalization of RT-qPCR results. The sequences of primers designed by NCBI Primer Blast are shown in Supplementary Table S1. Three independent replicates were conducted for this experiment. The fold change was calculated by 2−*ΔΔ*Ct.

## 3. Results

### 3.1. Cell Viability of H9C2 Cells Exposed to 3-T1AM

**Figure 1:**
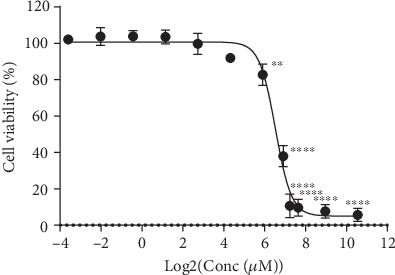
Cell viability was analyzed by CCK-8 of H9C2 cells with the treatment of different concentrations of 3-T1AM. Values are mean + SD of sextuple replicates. ^∗^*P* < 0.05, ^∗∗^*P* < 0.01, and ^∗∗∗^*P* < 0.001.

The results were obtained in H9C2 cells exposed to 120, 60, 20, 6.67, 2.22, 0.74, 0.24, and 0.08 *μ*M 3-T1AM and 3-T1AM-free growth medium. Our data indicated that the dose of 101.5 *μ*M 3-T1AM decreased the cell viability of H9C2 nearly 50% after 24 h treatment (Figure 1). Hence, 20 *μ*M 3-T1AM, which could not affect H9C2 cell viability, was used for the following experiments.

### 3.2. Illumina Sequencing and Preprocessing of Raw Data

RNA sequencing was performed on poly(A)-enriched RNA extracted from H9C2 cells and H9C2 cells treated with 3-T1AM. The raw data of sequencing was evaluated and is shown in Table 1. The Q30 data, the probability of an error base call 1 in 1000 times, were over 91%. The proportion of Q20, the probability of an error base call in 1 in 100 times, was more than 96%, which suggests that the quality of sequencing data was appropriated for the following data analysis.

### 3.3. Identification of Differentially Expressed Genes Associated with 3-T1AM Treatment

**Figure 2:**
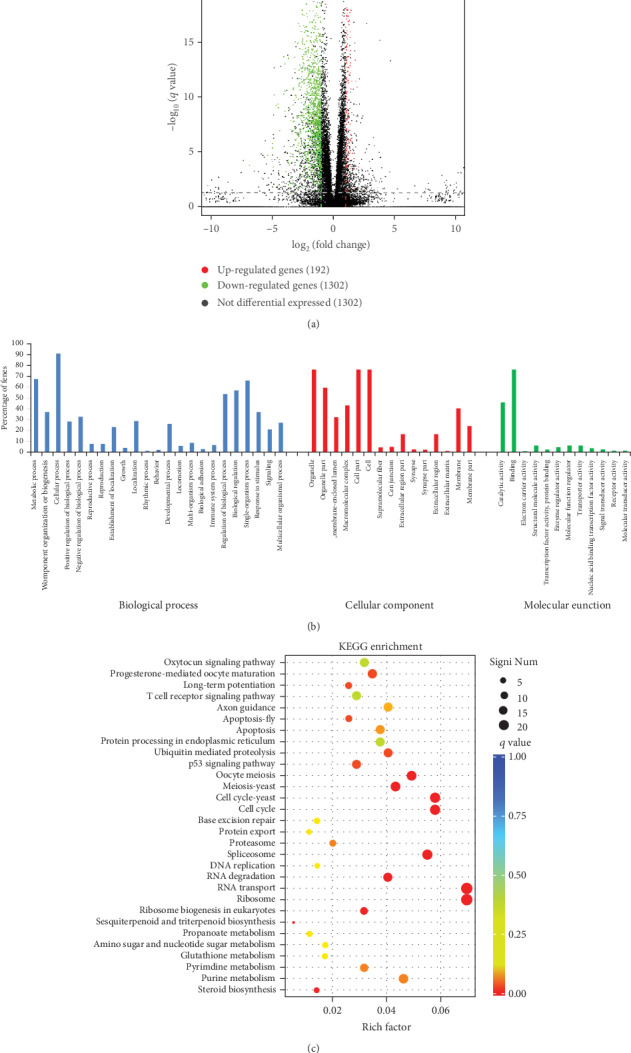
Functional analysis of the 1494 DEGs. (a) Volcano plots of significant differential expression of genes. The *x*-axis represents Log2(fold change), and the *y*-axis represents -Log10(*P* value). Red dots are Log2 > 1 and *P* value < 0.05. Green dots are Log2 < −1 and *P* value < 0.05. (b) Analysis of GO enrichment for the DEGs. The green colors represent significant biological process. The blue colors represent significant cellular component, and the yellow colors represent significant molecular function. (c) KEGG enrichment analysis for the DEGs. The pathways of significant differentially expressed genes are enriched.

The transcriptome profiles of H9C2 cells treated with 3-T1AM were compared with those of H9C2 cells. Differently expressed genes (DEGs) were identified according to Log2 fold changes and *P* value. Volcano plots were used to analyze DEGs, in which red spots and green spots represent upregulated and downregulated genes separately (Figure 2(a)). 1494 DEGs, including 192 upregulated and 1302 downregulated genes, were determined in the 3-T1AM group in comparison to the control group.

### 3.4. Analysis of Gene Ontology (GO) and Kyoto Encyclopedia of Genes and Genomes (KEGG) Pathway Enrichment

GO enrichment analysis was carried out to define the biological function of the 1494 DEGs. It is divided into three subontologies: biological process (BP), cellular component (CC), and molecular function (MF). In biological process, terms such as cellular process, metabolic process, and single-organism process (GO:0008152, GO:0071840, GO:0009987, GO:0048518, GO:0048519, GO:0022414, GO:0000003, GO:0051234, GO:0040007, GO:0051179, GO:0048511, GO:0007610, GO:0032502, GO:0040011, GO:0051704, GO:0022610, GO:0002376, GO:0050789, GO:0065007, GO:0044699, GO:0050896, GO:0023052, and GO:0032501) were significantly enriched. In the cellular component subontology, terms related to cell, cell part, and organelle (GO:0043226, GO:0044422, GO:0031974, GO:0032991, GO:0044464, GO:0005623, GO: 0099512, GO:0030054, GO:0044421, GO:0045202, GO:0044456, GO:0005576, GO:0031012, GO:0016020, and GO:0044425) were significantly enriched. In the molecular function subontology, terms including binding, catalytic activity, and structural molecular activity (GO:0003824, GO:0005488, GO:0009055,GO:0005198, GO:0000988, GO:0030234, GO:0098772, GO:0005215, GO:0001071, GO:0004871, GO:0004872, and GO:0060089) were significantly enriched (Figure 2(b)).

KEGG enrichment analysis was performed to identify relative pathway in which the 1494 DEGs were involved. A scatter plot was used to depict significantly enriched pathway terms. Pathways such as RNA transport, P53 signaling pathway, and ribosome were most significantly enriched terms in H9C2 cells treated with 3-T1AM compared with the control group(Figure 2(c)).

### 3.5. Disease Connectivity Analysis

We conducted an additional analysis of the DEGs using the Comparative Toxicogenomics Database (CTD), which permits the development of novel hypotheses about the relationships between 3-T1AM and cardiovascular diseases. To reduce interference of unrelated genes and identify the cardiovascular disease-associated genes, 90 genes were determined as hub genes (Table 2). In addition, we also verified that 68 genes were associated with heart diseases, 7 genes were associated with MELAS syndrome, 50 genes were associated with vascular diseases, and 22 genes were associated with cardiomyopathies.

### 3.6. PPI Network Construction

**Figure 3:**
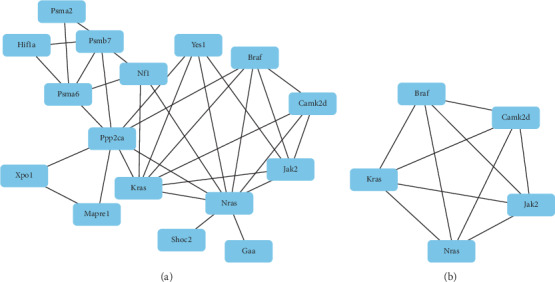
Hub genes and module analysis of these hub genes. (a) The PPI network of the 16 hub genes was identified (confidence score > 0.9). The nodes represent proteins, and the edges represent interactions. (b) The significant network module is composed of five nodes and 10 edges extracted from the PPI network (MCODE score = 4).

In order to further analyze a protein-protein interaction network of these 90 DEGs associated with cardiovascular diseases, a PPI network was generated based on the STRING database and Cytoscape 3.4.0 software. Our results indicated that a module that consisted of 16 nodes was identified from 90 genes associated with cardiovascular diseases (Figure 3(a)). By using the MCODE analysis, the module that consisted of 5 nodes and 10 edges (MCODE score = 4) was screened out (Figure 3(b)).

### 3.7. Verification of Differentially Expressed Genes by RT-qPCR

**Figure 4:**
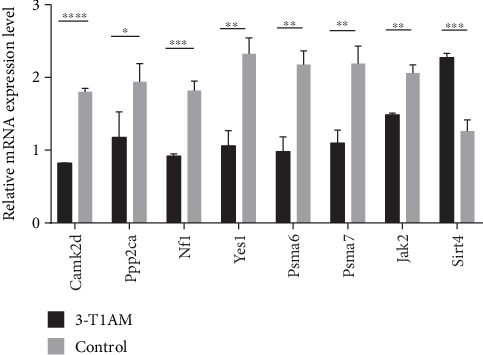
RT-qPCR validation of the RNA-Seq results. The results indicated that variation tendencies between the RNA-Seq and RT-qPCR data were identical. Values are mean ± SD of triplicate replicates.

To verify the reliability of the RNA-Seq results, eight cardiovascular-related candidates were chosen in both groups to verify differential expression. The results strengthened that variation tendencies between the RNA-Seq and RT-qPCR data were identical, verifying the reliability of the RNA-Seq data (Figure 4).

## 4. Discussion

Previous studies have indicated that 3-T1AM is an effective metabolite product of the TH that can decrease the heart rate and lower the cardiac output [16–18]. We speculated that cardiomyocytes would be an essential target site for 3-T1AM. RNA-Seq profile analysis is an efficacious assay for uncovering the potential molecular mechanisms of 3-T1AM and has been extensively accepted as a method to find the therapeutic targets of drugs [19]. In our investigation, we explored the DEGs and mechanisms of cultured H9C2 cell lines after 3-T1AM treatment using RNA-Seq and appropriate transcriptome analysis.

Our research substantiated that 3-T1AM can reduce the cell viability of H9C2 cells in a time- and dose-dependent manner using a CCK8 assay. Studies showed that doses of 3-T1AM used in in vitro studies were different in various cell lines [3, 20–22]. Then, in our study, 20 *μ*M 3-T1AM that could not affect cell viability after treatment for 24 h was chosen for RNA-Seq. A limitation of our study is that time- and concentration-related responses cannot be monitored according to the results of RNA-Seq.

A total of 1494 DEGs were identified in cultured H9C2 cells exposed to 3-T1AM for 24 h based on the screening criteria of DEGs in our study. Among them, only 90 DEGs associated with cardiovascular diseases were identified. To confirm the reliability of the RNA-Seq results, a number of DEGs, such as Camk2d, Ppp2ca, Nf1, Yes1, Psmb7, Sirt4, and Jak2, were selected and tested by RT-qPCR, which indicated that these two methods were in good agreement.

The comparative analysis of DEGs provides the therapeutic intervention for the cardiovascular diseases and delineates novel insight into other diseases. Several differentially expressed genes identified in this study, including HMGB1, ROCK2, and Jak2, are amenable in principle to diagnostic biomarkers and therapeutic targets. Previous studies indicated that upregulation of HMGB1 aggravated mechanical stress-induced cardiomyocyte hypertrophy via the RAGE/ERK1/2 signaling pathway [23]. Increased ROCK2 was associated with the pathogenesis of Ang-II-induced cardiac hypertrophy via regulating FHOD3 phosphorylation [24]. Moreover, upregulation of Jak2 activated the Jak2-STAT3 pathway, thereby alleviating myocardial ischemia-reperfusion injury, cardiomyocyte apoptosis, and ROS production in vitro [25].

It is widely known that 3-T1AM exerts a physiological function of lowering body temperature in rodents. Studies suggest that 3-T1AM acts through sirtuin-mediated pathways to metabolically reprogram fatty acid and glucose metabolism possibly through small-molecule signaling [26]. To understand biological functions of the 3-T1AM-induced DEGs in H9C2 cells, several GO terms which are related to metabolism were explored including the biological process category “metabolic function” and the molecular function category “catalytic activity”. In addition, glutathione metabolism, pyrimidine metabolism, and purine metabolism were found by KEGG analysis. These finding suggested that 3-T1AM could affect cardiomyocytes by metabolic function.

Previous studies found that hormone-binding domain was constituted by two binding sites, one of which interacts with thyroid receptors, resulting in a decreased transcriptional activity of numerous proteins via phosphorylate p53 [27]. Moreover, the p53 signaling pathway was also found by KEGG analysis of 3-T1AM-induced DEG. These results indicated that 3-T1AM is involved in the p53 signaling pathway, but whether it is involved in the p53 pathway needs to be further studied.

PPI network analysis sketched detailed interactions among the identified 90 DEGs associated with cardiovascular diseases. Furthermore, the top 10 hub genes for 3-T1AM treatment appeared to be Nras, Ppp2ca, Kras, Braf, Jak2, Psma6, Psmb7, Camk2d, Nf1, and Yes1. Five of these genes, Camk2d, Kras, Braf, JAK2, and Nras, were confirmed by the most significant module. Moreover, these genes were involved in KEGG pathways, such as the ErbB signaling pathway (rno04012), EGFR tyrosine kinase inhibitor resistance (rno01521), and chemokine signaling pathway (rno04062).

## 5. Conclusion

In conclusion, we identified 1494 DEGs in H9C2 cells for 3-T1AM treatment in our research. Of these, 90 genes had associated with cardiovascular diseases. Interestingly, we further employed comparative analysis of biological functions, related signaling pathways, and PPI network of these identified DEGs. The 5 hub genes in cardiovascular diseases were identified according to the PPI network analysis. In addition, our results provide a new insight into the therapeutic intervention of 3-T1AM for the cardiovascular diseases.

## Figures and Tables

**Table 1:** Raw data of 8 samples.

	3-T1AM (1)	3-T1AM (2)	3-T1AM (3)	3-T1AM (4)	Control (1)	Control (2)	Control (3)	Control (4)
Total read count (#)	58597112	53285564	48765512	55471208	56286724	58864010	62022768	55517916
Total base count (bp)	8402286096	7624535664	6982859772	7964658512	8055339995	8429665566	8909078782	7930285919
Average read length (bp)	143.39	143.09	143.19	143.58	143.11	143.21	143.64	142.84
Q10 base count (bp)	8398821444	7621323425	6979833944	7961065467	8052382833	8426272761	8905846091	7927563532
Q10 base ratio (%)	99.96%	99.96%	99.96%	99.95%	99.96%	99.96%	99.96%	99.97%
Q20 base count (bp)	8324251465	7552342304	6915469817	7888692485	7982828699	8350503900	8832549267	7861327327
Q20 base ratio (%)	99.07%	99.05%	99.03%	99.05%	99.10%	99.06%	99.14%	99.13%
Q30 base count (bp)	8081550131	7329798418	6707210805	7653233962	7758822935	8107333264	8595387916	7648155397
Q30 base ratio (%)	96.18%	96.13%	96.05%	96.09%	96.32%	96.18%	96.48%	96.44%
*N* base count (bp)	32774	29918	26862	30624	32389	33409	32739	31415
*N* base ratio (%)	0.00%	0.00%	0.00%	0.00%	0.00%	0.00%	0.00%	0.00%
GC base count (bp)	4724097515	4287982429	3977418356	4602592938	4353071110	4644560391	4811453986	4168979108
GC base ratio (%)	56.22%	56.24%	56.96%	57.79%	54.04%	55.10%	54.01%	52.57%

**Table 2:** The 90 enriched genes associated with cardiovascular diseases analyzed with the Comparative Toxicogenomics Database.

Disease name	Disease categories	*P* value	Corrected *P* value	Annotated gene quantity	Annotated genes	Genome frequency
Cardiovascular diseases	Cardiovascular disease	2.81*E*-13	3.78*E*-10	90	ANXA1|ATP7A|BRAF|CALM2|CALU|CAMK2D|CANX|CASP12|CASQ2|CCL2|COX1|COX2|COX3|CRBN|CXCL1|CYTB|des|DNM1L|EIF2A|ERAP1|FKBP14|FKTN|GAA|GATAD1|GLRX3|GSTA4|GSTT1|HIF1A|HMGB1|HMOX1|HSF2|HSPA9|HSPD1|JAK2|KLF13|KRAS|MAOA|MAPRE1|MEX3C|MFF|NCL|ND1|ND5|ND6|NDUFS1|NEDD4|NEXN|NF1|NR3C1|NR4A1|NRAS|NUCKS1|NUP155|PAK2|PDIA3|PON2|PPP2CA|PPP3R1|PROS1|PSEN2|PSMA2|PSMA6|PSMB7|PTP4A2|RASA1|RENBP|ROCK2|SCN1B|SGCA|SGCD|SHOC2|SIRT4|SLC4A3|STK39|TAP1|TBCA|TFPI|TFRC|TLL1|TNFAIP6|TRIM63|TRPM4|UFD1|UGCG|USP34|WDPCP|WDR12|WNK4|XPO1|YES1	1452/43293 genes: 3.35%

Heart diseases	Cardiovascular disease	7.42*E*-11	9.97*E*-08	68	BRAF|CALM2|CALU|CAMK2D|CANX|CASP12|CASQ2|CCL2|COX1|des|DNM1L|EIF2A|FKTN|GAA|GATAD1|GLRX3|HIF1A|HMGB1|HMOX1|HSF2|HSPA9|HSPD1|JAK2|KLF13|KRAS|MAPRE1|MEX3C|MFF|NCL|NDUFS1|NEDD4|NEXN|NF1|NR3C1|NR4A1|NRAS|NUCKS1|NUP155|PAK2|PDIA3|PPP2CA|PPP3R1|PSEN2|PSMA2|PSMA6|PSMB7|PTP4A2|RENBP|ROCK2|SCN1B|SGCA|SGCD|SHOC2|SIRT4|SLC4A3|TAP1|TBCA|TFRC|TLL1|TRIM63|TRPM4|UFD1|UGCG|USP34|WDPCP|WDR12|XPO1|YES1	1066/43293 genes: 2.46%

MELAS syndrome	Cardiovascular disease |Genetic disease (inborn) |Metabolic disease |Musculoskeletal disease |Nervous system disease	1.20E-06	0.00161	7	COX1|COX2|COX3|CYTB|ND1|ND5|ND6	22/43293 genes: 0.05%

Vascular diseases	Cardiovascular disease	2.59E-06	0.00348	50	ANXA1|ATP7A|CAMK2D|CANX|CASP12|CCL2|COX1|COX2|COX3|CRBN|CXCL1|CYTB|DNM1L|EIF2A|ERAP1|FKBP14|GSTA4|GSTT1|HIF1A|HMGB1|HMOX1|HSPA9|HSPD1|JAK2|KLF13|KRAS|MAOA|MAPRE1|MFF|NCL|ND1|ND5|ND6|NEDD4|NR3C1|NR4A1|PDIA3|PPP2CA|PPP3R1|PROS1|PSMA6|RASA1|STK39|TAP1|TFPI|TFRC|TNFAIP6|UGCG|WDR12|WNK4	917/43293 genes: 2.12%

Cardiomegaly	Cardiovascular disease |Pathology (anatomical condition)	3.31E-06	0.00444	17	CASQ2|des|FKTN|GAA|GATAD1|GLRX3|HIF1A|HMOX1|NEXN|PPP3R1|PSEN2|RENBP|ROCK2|SGCD|SIRT4|TRIM63|WDR12	168/43293 genes: 0.39%

Cardiomyopathies	Cardiovascular disease	5.17E-06	0.00695	22	CASP12|CASQ2|CCL2|COX1|des|FKTN|GAA|GATAD1|HIF1A|HMGB1|HSPD1|NDUFS1|NEXN|PSEN2|RENBP|SGCA|SGCD|SIRT4|TBCA|TFRC|TRIM63|WDR12	271/43293 genes: 0.63%

Cerebral small vessel diseases	Cardiovascular disease |Nervous system disease	5.56E-06	0.00747	7	COX1|COX2|COX3|CYTB|ND1|ND5|ND6	27/43293 genes: 0.06%

## Data Availability

The data used to support the findings of this study are available from the corresponding author upon request.

## Supplementary Materials

Supplementary Materials Supplemental Data Table S1: primers used for RT-qPCR.
