# Supplementary material for: Comparative Transcriptome Analysis Reveals the Potential Cardiovascular Protective Targets of the Thyroid Hormone Metabolite 3-Iodothyronamine (3-T1AM)
Source: Biomed Res Int. 2020 Jun 19;2020:1302453. doi: 10.1155/2020/1302453 (PMC7322601; doi:10.1155/2020/1302453)
Supplement: Supplementary Materials — Supplemental Data Table S1: primers used for RT-qPCR. [file 1302453.f1.docx]

Supplemental Data Table S1 Primers used for RT-qPCR
